# Phylogenetic Reconstruction by Cross-Species Chromosome Painting and G-Banding in Four Species of Phyllostomini Tribe (Chiroptera, Phyllostomidae) in the Brazilian Amazon: An Independent Evidence for Monophyly

**DOI:** 10.1371/journal.pone.0122845

**Published:** 2015-03-25

**Authors:** Talita Fernanda Augusto Ribas, Luis Reginaldo Ribeiro Rodrigues, Cleusa Yoshiko Nagamachi, Anderson José Baia Gomes, Jorge das Dores Rissino, Patricia Caroline Mary O'Brien, Fengtang Yang, Malcolm Andrew Ferguson-Smith, Julio Cesar Pieczarka

**Affiliations:** 1 Laboratório de Citogenética, ICB, Universidade Federal do Pará, Belém, Pará, Brazil; 2 Laboratório de Genética e Biodiversidade, ICED, Universidade Federal do Oeste do Pará, Santarém, Pará, Brazil; 3 CNPQ Researcher, Belém, Pará, Brazil; 4 Cambridge Resource Centre for Comparative Genomics, University of Cambridge, Cambridge, United Kingdom; 5 Cytogenetics Facility, Wellcome Trust Sanger Institute, Cambridge, United Kingdom; Onderstepoort Veterinary Institute, SOUTH AFRICA

## Abstract

The subfamily Phyllostominae comprises taxa with a variety of feeding strategies. From the cytogenetic point of view, Phyllostominae shows different rates of chromosomal evolution between genera, with *Phyllostomus hastatus* probably retaining the ancestral karyotype for the subfamily. Since chromosomal rearrangements occur rarely in the genome and have great value as phylogenetic markers and in taxonomic characterization, we analyzed three species: *Lophostoma silvicola* (LSI), *Phyllostomus discolor* (PDI) and *Tonatia saurophila* (TSA), representing the tribe Phyllostomini, collected in the Amazon region, by classic and molecular cytogenetic techniques in order to reconstruct the phylogenetic relationships within this tribe. LSA has a karyotype of 2n=34 and FN=60, PDI has 2n=32 and FN=60 and TSA has 2n=16 and FN=20. Comparative analysis using G-banding and chromosome painting show that the karyotypic complement of TSA is highly rearranged relative to LSI and PHA, while LSI, PHA and PDI have similar karyotypes, differing by only three chromosome pairs. Nearly all chromosomes of PDI and PHA were conserved *in toto*, except for chromosome 15 that was changed by a pericentric inversion. A strongly supported phylogeny (bootstrap=100 and Bremer=10 steps), confirms the monophyly of Phyllostomini. In agreement with molecular topologies, TSA was in the basal position, while PHA and LSI formed sister taxa. A few ancestral syntenies are conserved without rearrangements and most associations are autapomorphic traits for *Tonatia* or plesiomorphic for the three genera analyzed here. The karyotype of TSA is highly derived in relation to that of other phyllostomid bats, differing from the supposed ancestral karyotype of Phyllostomidae by multiple rearrangements. Phylogenies based on chromosomal data are independent evidence for the monophyly of tribe Phyllostomini as determined by molecular topologies and provide additional support for the paraphyly of the genus *Tonatia* by the exclusion of the genus *Lophostoma*.

## Introduction

Subfamily Phyllostominae Gray, 1825, includes taxa that have diversified feed strategies, including carnivorous, strictly insectivorous and a combination of frugivorous and insectivorous [1,2]. They are widely distributed throughout the Neotropics, extending from southern United States, into southern Brazil and some major islands off the South America coast [3,4]. The different genera of this subfamily represent relatively antique lineages that diverged in the Early to Mid-Miocene at approximately 18.6–19.5 million years ago (MYA) [5].

Phyllostominae bats represent a taxonomically controversial group. Although phylogenetic analyses of morphology [6], alloenzymes [7], immunologic distances [8], chromosomes [9–11], and molecules [5,12], have been made to clarify the relationships among these and other genera, their relationships are still unclear, with different data sets leading to different phylogenies and classifications [12–17].

Baker et al. [15] proposed a classification with nine genera grouped into three tribes for the subfamily Phyllostominae: *Lophostoma*, *Mimon*, *Phylloderma*, *Phyllostomus*, *Tonatia* (Phyllostomini), *Macrophyllum*, *Trachops* (Macrophyllini), *Chrotopterus* and *Vampyrum* (Vampyrini). This arrangement was recently supported by Hoffmann et al. [5]. The genera *Macrotus*, *Micronycteris*, *Lampronycteris*, *Lonchorhina*, *Trinycteris* and *Glyphonycteris* which were classified within Phyllostominae according to Koopman [18], McKenna and Bell [19], Simmons and Voss [6], Wetterer et al. [16], and Jones et al. [20], were removed and classified in another subfamily.

Within Phyllostomini, Goodwin [21] and Genoways and Williams [22] reviewed the genus *Tonatia* using morphological characters. The monophyly of *Tonatia* based on morphological characters was questioned by Arnold et al. [7] and by Honeycutt and Sarich [8], because albumin immunologic distances between *T*. *bidens* and other species of *Tonatia* were equally as great as between *T*. *bidens* and *Phyllostomus*. In the most recent review, Lee et al. [12] classified all the species previously included in *Tonatia*, except *T*. *bidens* and *T*. *saurophila*, to the genus *Lophostoma*. This same arrangement was found by Porter et al. [17] in their analysis of nuclear and mitochondrial genes.

From the chromosomal point of view, previous studies in Phyllostomini showed a high variation in diploid number with 2n = 16 for *Tonatia* [9,23], and 2n = 34 for *Lophostoma silvicola* [11, 24–25]. Phyllostomini have different rates of karyotype evolution, from the chromosomal conservatism observed in the genera *Phyllostomus*, *Mimon*, *Phylloderma*, and in some species of *Lophostoma*, to the species with a high degree of karyotypic evolution as found in the karyotypes of *Tonatia bidens*, *T*. *saurophila* and *Lophostoma schulzi* [9–10,26]. Based on classical cytogenetics, Patton and Baker [9] proposed that at least 20 types of chromosomal rearrangements were responsible for the highly divergent karyotype found in the genus *Tonatia*, although the homology of chromosome arms between *Tonatia* and other genera cannot suggest certainty based solely on G-banding.

In this regard, chromosome painting has contributed to the elucidation of chromosomal homologies between species phylogenetically distant [27–32], or between species with highly rearranged karyotypes observed in other vertebrates [33–37]. Thus, chromosome painting has proved to be an excellent tool to detect and analyze the chromosomal changes that have occurred in the evolutionary history of taxa with megaevolved karyotypes, as in the genus *Tonatia* [38].

Until now, seven species in three phyllostomid subfamilies were studied by cross-species chromosome painting using probes of *Phyllostomus hastatus* and *Carollia brevicauda* [39]: *Dhyphylla eucaudata*, *Diaemus youngi*, *Desmodus rotundus* (Desmodontinae) [40], *Artibeus obscurus*, *Uroderma bilobatum*, *U*. *magnirostrum* (Stenodermatinae) [41], *Micronycteris hirsuta* (Micronycterinae) [36]. Here, we analyzed three species of subfamily Phyllostominae, *Tonatia saurophila*, *Lophostoma silvicola* and *Phyllostomus discolor* from the Amazon rainforest (Brazil), by cross-species chromosome painting using chromosome-specific probes from *Carollia brevicauda* and *Phyllostomus hastatus*. We used all Desmodontinae bats as the phylogenetic outgroup [40], and compared our results with those obtained by Pieczarka et al. [41]. Our aim was to establish reliable comparative chromosome maps between these species and use this dataset for the reconstruction of chromosomal phylogenies within the tribe Phyllostomini.

## Results

### Tonatia saurophila

**Fig 1:**
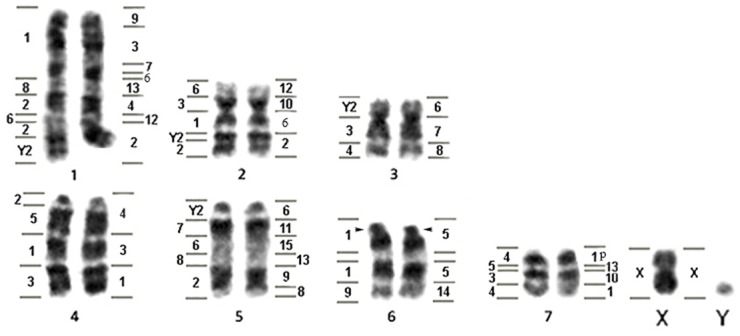
*Tonatia saurophila* G-banded karyotype showing homology to *Carollia brevicauda* (left) and *Phyllostomus hastatus* (right) chromosomes.

The results show that all nine *Tonatia saurophila* have 2n = 16 and FN = 20, with three pairs being bi-armed and four one-armed chromosomes pairs; the X is a medium-sized submetacentric and the Y a small acrocentric (Fig 1). Constitutive heterochromatin (CH) was observed in small amounts and restricted to the pericentromeric regions of all chromosomes (data not shown). AgNO_3_-staining and FISH with 18S rDNA probes revealed a Nucleolar Organizer Region (NOR) in the distal region of the short arm of pair 6 (Fig 1).

ZOO-FISH analyses using whole chromosome probes of *Phyllostomus hastatus* (PHA) revealed 32 homologous segments on the *Tonatia saurophila* (TSA) genome (Fig 1). Four paints of *Phyllostomus* (PHA-11, PHA-14, PHA-15 and X), showed only one fluorescent signal in the chromosomes of *Tonatia*, corresponding to segments of TSA-5 (PHA-11 and 15), TSA-6, and whole TSA-X. PHA-5 also hybridized to only one chromosome, the pair 6 of TSA, but in early metaphase with much distended chromosomes, presented two hybridization signals, separated by a region without homology. No other PHA or CBR probe hybridized in this region.

Eight PHA probes gave two fluorescent signals, with each probe marking two distinct chromosomes in *Tonatia*: PHA-2 (TSA-1 and 2), PHA-3 (TSA-1 and 4), PHA-4 (TSA-1 and 4), PHA-7 (TSA-1 and 3), PHA-8 (TSA-3 and 5), PHA-9 (TSA-1 and 5), PHA-10 (TSA-2 and 7), PHA-12 (TSA-1 and 2). PHA-13 yielded **three** hybridization signals in TSA-1, 5 and 7, while PHA-1 showed **three** signals, but hybridized to two distinct chromosomes: TSA-4 and 7. PHA-6 showed four hybridization signals, corresponding to four chromosomes on TSA-1, 2, 3 and 5.

Hybridization of *Carollia brevicauda* (CBR) whole chromosome probes revealed 31 homologous segments on the *Tonatia saurophila* (TSA) genome (Fig 1). Three paints of CBR gave one hybridization signal each on the karyotype of TSA: CBR-7 (TSA-5), CBR-9 (TSA-6) and CBR-X (TSA-X). Two paints of CBR hybridized in two *Tonatia* chromosomes: CBR-8 (TSA-1 and 5) and CBR-5 (TSA-4 and 7), while CBR-4 probes also hybridized to two chromosomes of *Tonatia*: TSA-3 and 7, but showed three hybridization signals.

CBR-6 hybridized to chromosomes TSA-1, 2 and 5, and showed one fluorescent signal on each. CBR-3 gave four signals on four distinct chromosomes of TSA-2, 3, 4 and 7. CBR-2 showed five hybridization signals, but on just four chromosomes on TSA: 1, 2, 4 and 5. CBR-1 hybridized to chromosomes TSA-1, 2, 4 and 6, but showed five markings, because in TSA-6 it shows two hybridization signals, in the same way as in PHA-5. CBR-Y2 showed four signals to four chromosomes of TSA-1, 2, 3 and 5 (Fig 1).

### Lophostoma silvicola

**Fig 2:**
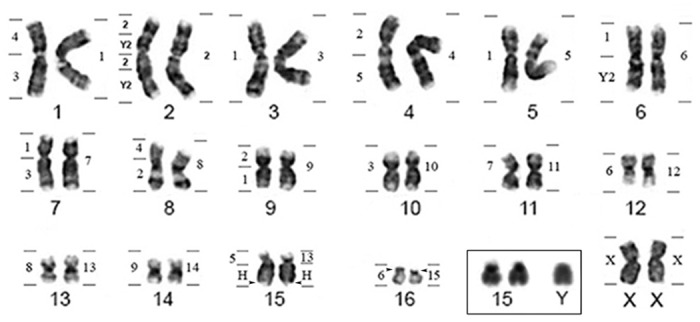
*Lophostoma silvicola* G-banded karyotype showing homology to CBR (left) and PHA (right) chromosomes. Box: heterochromatic pairs 15 and Y.

*Lophostoma silvicola* has a karyotype of 2n = 34 and FN = 60, including 28 bi-armed and 4 one-armed chromosomes; the X is metacentric and the Y acrocentric (Fig 2). CH is located in the pericentromeric region of all chromosomes and in the whole long arm of acrocentric chromosomes 15 and Y. Staining with AgNO_3_ and FISH with 18S rDNA probes revealed a Nucleolar Organizer Region (NOR) in the distal portion of the long arm of chromosome 15 and in the short arm of pair 16 (Fig 2).

Comparative painting with *Phyllostomus hastatus* (PHA) probes revealed 17 homologous segments on the *Lophostoma silvicola* (LSI) genome. Most PHA probes correspond to whole chromosomes in karyotype of LSI and only PHA-13 showed two signals, hybridizing fully to LSI-13 and to the short arm of chromosome LSI-15 (Fig 2).

Hybridization of *Carollia brevicauda* (CBR) whole chromosome probes revealed 26 homologous segments on the *Lophostoma silvicola* (LSI) genome. The number of homologous segments from CBR chromosomes in the karyotype of LSI extended from one to five. Four CBR probes hybridized to whole LSI chromosomes, with only one fluorescent signal in each: CBR-7 (LSI-11), CBR-8 (LSI-13), CBR-9 (LSI-14) and CBR-X.

**Fig 3:**
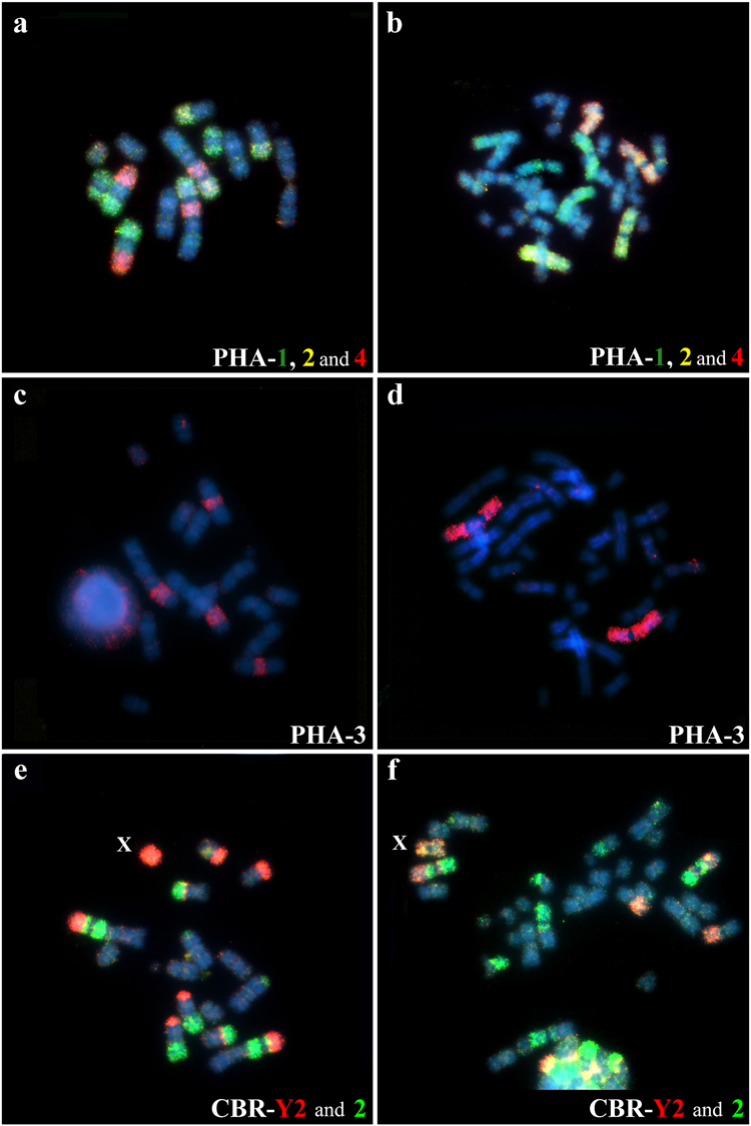
Chromosomal painting in *Tonatia saurophila* (a, c and e), *Phyllostomus discolor* (b) and *Lophostoma silvicola* (d and f), using PHA (above and middle) and CBR probes (below).

Three paints of CBR marking three different chromosomes on LSI, each gave two fluorescent signals: CBR-4 (LSI-1 and 8), CBR-5 (LSI-4 and 15) and CBR-6 (LSI-12 and 16). CBR-3 paint hybridized to three distinct chromosomes on LSI: 1, 7 and 10. CBR-Y2 showed three hybridization signals, but hybridized to just two chromosomes of LSI-2 and 6. CBR-1 showed four signals on chromosomes of LSI-3, 5, 6, 7. CBR-2 showed five signals, but hybridized to four chromosomes of LSI-2, 4, 8 and 9 (Figs 2 and 3). The Fig 3 shows hybridization with CBR and PHA whole probes on metaphases of TSA and LSI.

### Phyllostomus discolor

**Fig 4:**
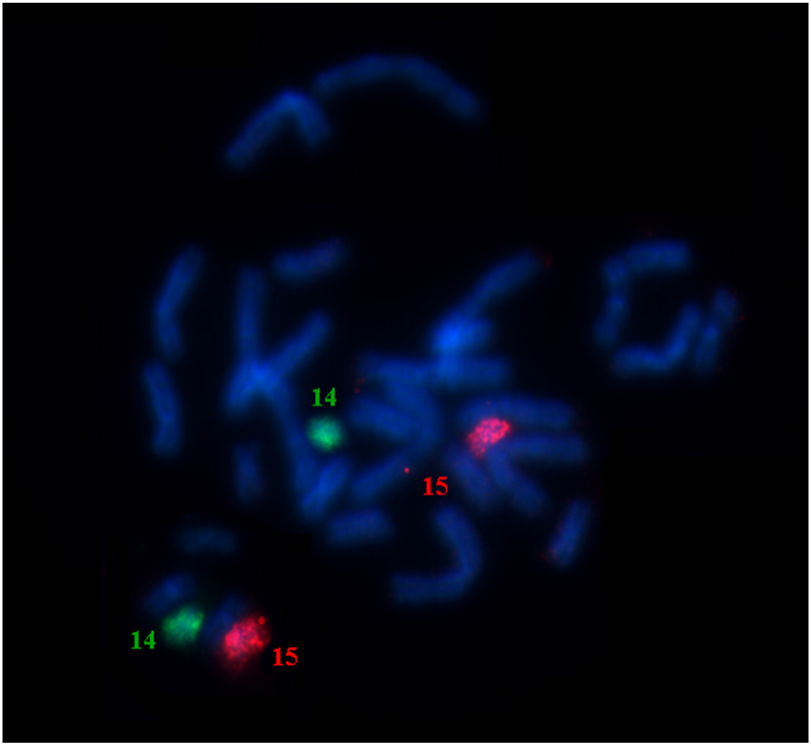
Whole chromosome probes FISH of PHA-14 (green) and PHA-15 (red) on PDI chromosomes, showing that PDI chromosomes are homologous to both PHA whole chromosomes their despite of metacentric form.

**Fig 5:**
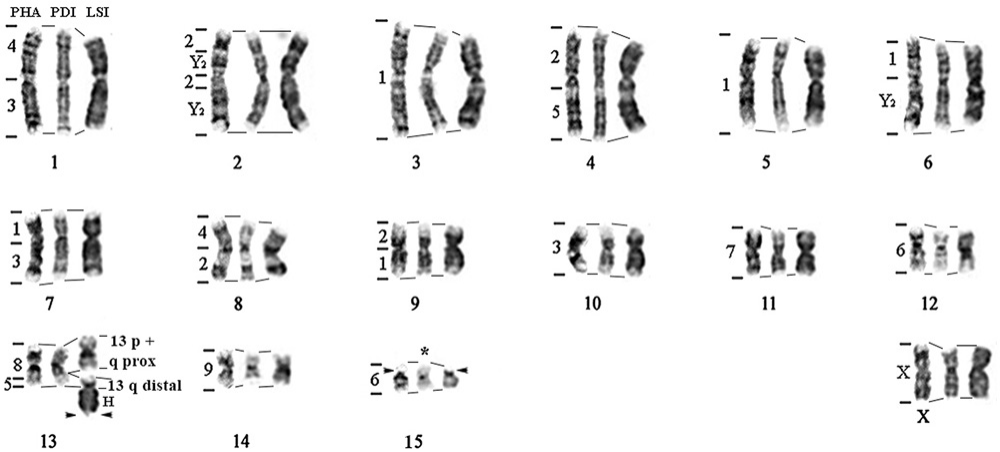
Comparative analysis using G-banded chromosomes of PHA, PDI and LSI. H = constitutive heterochromatin; NOR = Nucleolar Organizer Regions; *Pericentric inversion of pair 15 of PDI to PHA and LSI. Numbers on left: chromosomes from *Carollia brevicauda*. Numbers below: chromosomes from *Phyllostomus hastatus*.

*Phyllostomus discolor* has 2n = 32 and NF = 60, all chromosomes are bi-armed, except the Y that is acrocentric. CH is located in the pericentromeric region of all chromosomes. Staining with AgNO_3_ and FISH with 18S rDNA probes revealed a NOR in the distal portion of pair 15 (data not shown). Due to the highly conserved karyotype of PDI, when compared to PHA, we have only hybridized two whole chromosome paints corresponding to chromosomes PHA-14 and 15 which we presume to be involved in rearrangements, the latter corresponding to the acrocentric pair with a NOR inverted in PDI (Figs 4 and 5).

### Phylogenetic analyses in Phyllostomini

**Fig 6:**
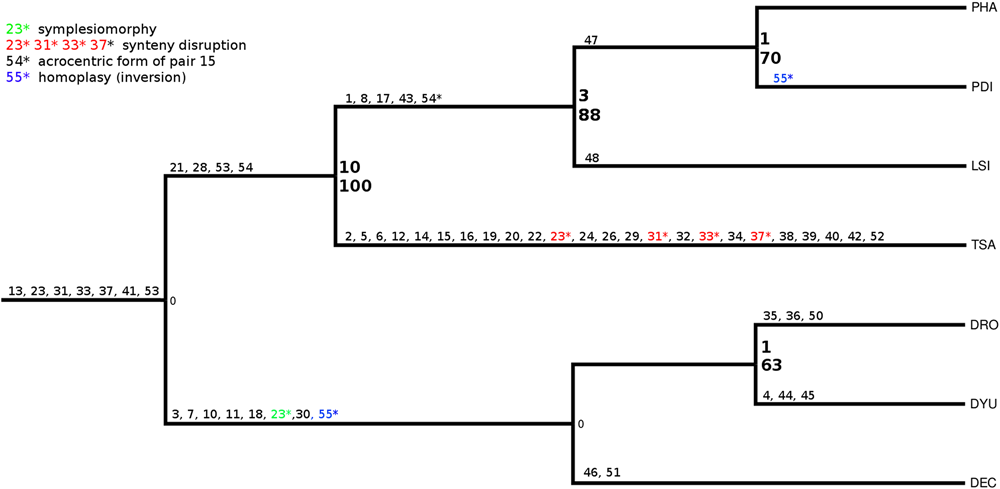
Cladogram obtained after the analysis by PAUP of the species PHA, PDI, LSI and TSA, using DEC, DYO, and DRO as outgroup using the chromosomal rearrangements as the unique caracters. Bold numbers indicate Bremer test (above) and bootstrap (below) values for each branch. The numbers refers to the chromosomal changes listed on the Basic Data Matrix (S1 Table). All the chromosomal changes (55) were mapped *a posteriori*, including 32 autapomorphies, 15 synapomorphies, 7 plesiomorphies and one homoplasy.

Maximum parsimony analysis resulted in a tree with score = 56 and consistency index = 0.9643. High bootstrap values support the monophyly of the Phyllostomini tribe. *Tonatia*, *Phyllostomus* and *Lophostoma* were grouped into a single branch, strongly supported (bootstrap = 100). *Tonatia* was in a basal position within Phyllostomini (bootstrap = 100 and Bremer = 10 steps), followed by a branch grouping *Lophostoma* and *Phyllostomus* (bootstrap = 88 and Bremer = 3 steps), and a group formed by *P*. *hastatus* and *P*. *discolor* that are sister taxa (bootstrap = 70 and Bremer = 1 step). Desmodontinae bats were used as outgroups and were grouped together with a polytomy (bootstrap = 63 and Bremer = 1) (Fig 6).

## Discussion

### Karyotypic variation in Phyllostomini

The karyotype of *Tonatia saurophila* is similar to those described for specimens from Central America with 2n = 16, FN = 20 [9, 22–23, 25, 42]. The karyotype of specimens of *Lophostoma silvicola* with 2n = 34, FN = 60 analyzed here is similar to those described by Gardner [24] and Baker et al. [43], for specimens of Ecuador and Suriname, suggesting high karyotype stability in *L*. *silvicola* despite its wide geographical distribution. The karyotype of *Phyllostomus discolor* is similar to those described for Central American specimens [9], Southeastern and North Brazil [44–47].

According to Patton and Baker [9] and Baker and Bickman [10], the karyotype of *T*. *saurophila* is so derived that no chromosomal arms proposed on the ancestral karyotype of phyllostomid could be identified by classical cytogenetics. Here, we identified chromosomal synteny using chromosome painting associated with G-banding. Some chromosomal segments previously suggest as plesiomorphic for Phyllostomidae [39–41], were found entire in *Tonatia* genome as PHA-14 (CBR-9), PHA-11 (CBR-7) and PHA-15 (CBR-6q distal). This result demonstrates the usefulness of chromosome painting for the identification of chromosomal homologies when classical cytogenetics is non-informative.

Most genera of phyllostomid bats have highly conserved karyotypes, but there are extensive karyotypic variations among some genera, which make intergeneric comparison by G-banding almost impossible [13, 41, 46, 48]. In contrast, it is possible to find chromosomal homeologies in all genera of Phyllostominae, except for *Tonatia* [9]. We found extensive similarity between the G-banding karyotypes of *Lophostoma silvicola*, *Phyllostomus hastatus*, and *P*. *discolor*. PHA has 2n = 32 and FN = 58, PDI also has 2n = 32 but FN = 60, while LSI has 2n = 34 and FN = 60 and differs from PHA only by three chromosome pairs (LSI-13, 15 and 16). PDI and PHA have all chromosomes without rearrangements, except for a pericentric inversion in chromosome 15 [46–47] (Fig 5).

Rodrigues et al. [46] suggest that the metacentric form of pair 15 arose in *Phyllostomus discolor* and *Mimon crenulatum* from a fusion between two pairs of acrocentric chromosomes homologous to *Macrotus waterhousii* chromosomes, one of them NOR-labeled. This hypothesis is supported by the distal position of the NOR-labeled chromosome shared between *Phyllostomus hastatus* and *Phylloderma stenops* (acrocentric form), and between *P*. *discolor* and *M*. *crenulatum* (metacentric form). So, according to these authors, *Phyllostomus discolor* presents the primitive form of chromosome 15 for the *Phyllostomus* genus shared with *Mimon*. On the other hand, our data show that the metacentric form is autapomorphic in *Phyllostomus discolor* and the acrocentric form is synapomorphic between *Lophostoma silvicola* and *P*. *hastatus* (Fig 5). We suggest that subsequent changes in the centromeric position of chromosome 15 as observed in other species of Phyllostomidae (eg., *Rhinophylla pumilio*, *Glossophaga soricina* and *Mimon crenulatum*, in Gomes et al. [47]), are possibly due to amplification of ribosomal DNA cistrons, accumulation of constitutive heterochromatin or centromeric repositioning. Evolutionary studies in various organisms clearly indicate that centromeric repositioning is not a rare event in karyotype evolution and should be considered when examining the evolution of chromosome structure [49].

An alternative explanation would be that the two forms of pair 15 are not related, having originated from ancestral chromosomal polymorphisms of Phyllostomini, with the fluctuations in size becoming fixed randomly in several branches (hemiplasy), as previously suggested for Chiroptera, where the presence of the chromosomal synteny HSA 1/6/5 is shared by one family (Pteropodidae) and one subfamily (Megadermatinae). This was formerly interpreted to be homoplasic [50] but might instead be an example of hemiplasy [51]. Another less parsimonious hypothesis would be that the form of metacentric chromosome pair 15 has evolved many times within Phyllostomidae through breakpoint reuse (i.e. true homoplasy) or different breakpoints, hence non not being strictly homology.

**Fig 7:**
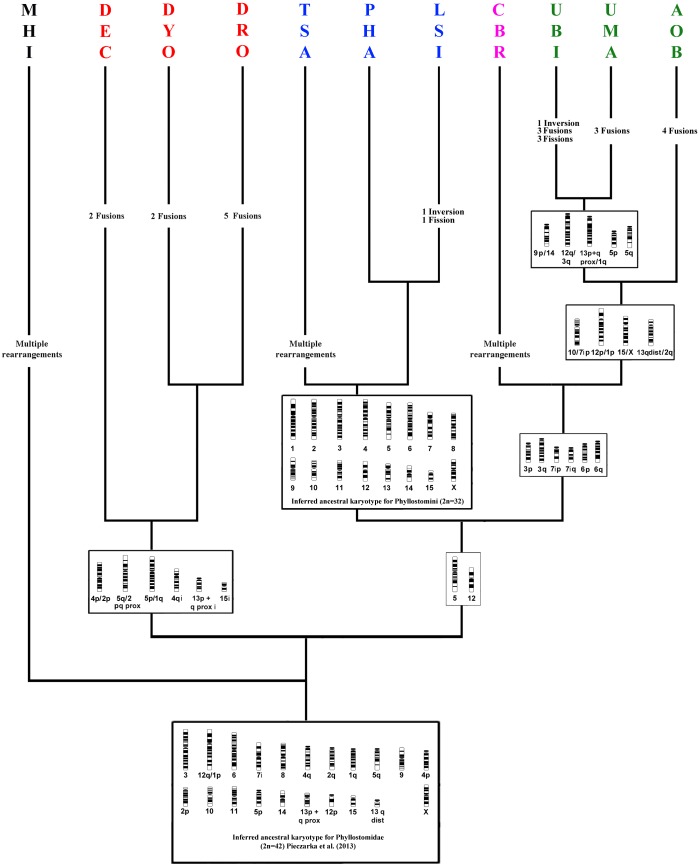
Partial reconstruction of chromosomal changes in phyllostomid bats based on Baker et al. [15] and Hoffmann et al. [5] phylogenies for subfamily Phyllostominae. The chromosome nomenclature followed Pieczarka et al. [39,41], and refers to homology with PHA chromosomes. Chromosome data for MHI are from Ribas et al. [36], for Desmodontinae are from Sotero-Caio et al. [40], and for PHA and CBR are from Pieczarka et al. [39].

Despite other chromosomal modifications between the compared species, we observe that chromosome PHA-13 was derived from fission as found in the putative ancestral karyotype of leafed-nose bats [41], in the Phyllostomini tribe (Fig 7), and possibly in the Phyllostominae subfamily. The fusioned form of this chromosome is shared between members of the genus *Phyllostomus*, and with *Mimon crenulatum* [47]. On the other hand, both on *Lophostoma silvicola* and *Tonatia saurophila* this chromosome is found on a fissioned way, but the fissioned segments were subsequently rearranged, with heterochromatin addition in *Lophostoma silvicola*. So, in both species this chromosome has a derivate morphology in regard of the ancestral state for the tribe.

### Phylogenetic relationships in Phyllostomini

We mapped by chromosome painting the karyotype of three species of phyllostomid bats: *Lophostoma silvicola*, *Phyllostomus discolor* and *Tonatia saurophila*, and compared the results among the Phyllostomidae species mapped so far: PHA, CBR [39], AOB, UBI, UMA [41], DEC, DRO and DYO [40], and MHI [36] (Fig 5). Our analysis expands the previous results on Phyllostomidae, confirming those results and making clearer the phylogenetic relationships within the family. Our results are independent confirmation of Baker et al. [15], (to Phyllostomidae) and Hoffmann et al. [5] (to Phyllostominae) molecular phylogenies.

The karyotype of TSA is highly rearranged relative to LSI and PHA. A few syntenies were conserved without rearrangement, consistent with the hypothesis of karyotypic megaevolution [10], and most associations were demonstrated to be autapomorphic traits for TSA or plesiomorphic (PHA-11, 14 and 15) traits for the three genera analyzed here and for other phyllostomid bats already mapped [39–41].

Pieczarka et al. [41] proposed a primitive karyotype for eight species belonging to four subfamilies of Phyllostomidae by chromosome painting and G–banding. Based on parsimony analyses they concluded that the 2n = 42, FN = 60 karyotype was most likely the primitive condition for the taxa mapped so far. Four synapomorphic chromosomes (PHA-1, 2, 4 and 7) for Phyllostominae were suggested based on *Phyllostomus hastatus* only. Here, we tested and confirmed this hypothesis by phylogenetic analysis associated with classical banding and chromosome painting (Figs 4 and 5), since LSI and PDI share these whole chromosomes and added PHA-13 chromosome as synapomorphic to the tribe. Therefore, we suggest a karyotype ancestral to the tribe Phyllostomini (Fig 7).

From G-banding analysis it is possible to assume that these four chromosomes are present and fully preserved in the karyotypes of *Mimon crenulatum* and *Phyllostomus discolor*, both members of Phyllostomini [9, 47] (Fig 5 for PDI). On the other hand, in the karyotype of *Tonatia saurophila* these chromosomes are rearranged. The validity of synapomorphies for the tribe Phyllostomini is viable only if we regard the chromosome form in *T*. *saurophila* as a derived condition. Considering the high level of karyotypic rearrangements found in this species, this is a plausible hypothesis (Figs 1 and 6). In TSA, PHA-7 is broken into two segments of different sizes, suggesting that chromosome breaks in TSA occurred from the ancestral submetacentric form, and is not inverted, as found in other Phyllostomini [9].

The putative primitive karyotype for the tribe Phyllostomini with 2n = 32 chromosomes (Fig 6), identical to the karyotype of *P*. *hastatus*, suggests that the *Tonatia* karyotype with 2n = 16 was derived, having arisen by multiple chromosomal rearrangements. In molecular phylogenies, and also in our chromosomal phylogeny, *Tonatia* was on a basal branch (Fig 7). In fact chromosome rearrangements do not behave in a clockwork fashion [10, 52–53], which is obvious also here where there is no correlation between primitive branching and a primitive karyotype.

The monophyly of the tribe Phyllostomini was supported by a high value of Bremer (10 steps), indicating strong robustness of the analysis (Fig 7). However, Bremer values for *Lophostoma* and *Phyllostomus* clade were relatively low (3), and the genus *Phyllostomus* clade was the lowest possible (1), but for a branch with a small number of characters parsimony-informative since the karyotypes are almost identical and similar to the primitive Phyllostomidae karyotype (meaning that the chromosome traits are plesiomorphic and are not taken into account in the cladistics analysis), even a low Bremer may be significant [54], since this test is qualitative and chromosomal rearrangements are considered to be rare events in the genome [55].

Analysis among genera of Phyllostominae shows that these lineages are relatively old, arising in the Mid-Miocene, and divergences among tribes happened in the Early Miocene. The node that gave rise to the genus *Tonatia* is 16.2 MYA, while *Tonatia* species arose 12.1 MYA, and the divergence of *Lophostoma* from the remainder of the Phyllostomini must have been at least 12–13 MYA [5]. This was at the time when differentiation of species with stable karyotypes (karyotypic stasis) occurred, as found in *Phyllostomus* and *Phylloderma*, and when radically reorganized karyotypes (karyotypic megaevolution) occurred in *Tonatia*. In *Lophostoma* there are species that have both types of karyotype evolution.

Although members of the *Tonatia* species group are similar morphologically to *Lophostoma silvicola* and distinguished only by width of the lower incisors, analyses of karyotypic and molecular data indicate that they are highly diverged from other species of *Lophostoma* [5,9,12,17]. Relationships of sister-taxon to *Phyllostomus* and *Tonatia*, or *Lophostoma* and *Tonatia* were inferred by molecular data, as well as the basal position of *Tonatia* to Phyllostomini [5,12,15,17]. Here, chromosomal phylogeny was consistent with the molecular topologies for Phyllostominae proposed by Lee et al. [12] and Hoffmann et al. [5], supporting the basal position of *Tonatia* for the Phyllostomini tribe and the relationship of sister-taxon between *Lophostoma* and *Phyllostomus*, due the one-armed chromosome 15 shared between these two taxa (Figs 4 and 5).

## Material and Methods

### Samples and metaphases

Three species were analyzed cytogenetically: *Tonatia saurophila* (TSA, five males and four females), *Lophostoma silvicola* (LSI, three males and six female) and *Phyllostomus discolor* (PDI, one female), from states of Pará, Amazonas and Mato Grosso, Brazil. Chromosomal preparations were obtained by direct extraction from bone marrow after Colchicine treatment following Baker et al. [56] and fibroblast cell culture following Moratelli et al. [57]. Cell lines were established in the Laboratório de Citogenética, Instituto de Ciências Biológicas, Universidade Federal do Para, Belem, Para, Brazil. The karyotypes of TSA were arranged according to Patton and Baker et al. [9], LSI according Honeycut et al. [25] and PHA according to Pieczarka et al. [39]. JCP has a permanent field permit, number 13248 from “Instituto Chico Mendes de Conservação da Biodiversidade”. The Cytogenetics Laboratory from UFPa has a special permit number 19/2003 from the Ministry of Environment for samples transport and 52/2003 for using the samples for research. The Ethics Committee (Comitê de Ética Animal da Universidade Federal do Pará) approved this research. Specimens were maintained in the lab with food and water, free from stress, until their necessary euthanasia, made with intraperitoneal injection of buffered and diluted barbiturates after local anesthetic.

### Chromosomal banding

Conventional staining was used for diploid (2n) and fundamental numbers (FN) determination. G-banding followed two distinct methods: trypsin treatment [58] and saline solution (2xSSC) incubation [59]. In both methods the metaphases were stained with Wright´s solution. C-banding was carried out according to Sumner [60] and Ag-NOR staining followed Howell and Black [61].

### Fluorescence *in Situ* Hybridization (FISH)

FISH with digoxigenin labeled telomeric probes (All Human Telomere Probes, Oncor) were performed according to the manufacturer’s protocol and 18S rDNA probes from *Prochilodus argenteus* [62] were labeled with biotin or digoxigenin by nick translation. Primary PCR products of whole sorted chromosomes from *P*. *hastatus* (PHA) and *C*. *brevicauda* (CBR) [39] were labeled either with biotin-16-dUTP (Boehringer Mannheim), fluorescein isothiocyanate-12-dUTP (Amersham), or Cy3-dUTP by taking 1μl of product to a second round of DOP-PCR using the same primer. The biotin probes were detected with avidin-Cy3 or avidin-FITC.

Chromosome painting was performed as previously described [39, 63]. Briefly, the slides were incubated in pepsin solution, and dehydrated in an ethanol series (70, 90 and 100%), air-dried and aged in a 65°C incubator for two hours. Chromosomal DNA was denatured in 70% formamide/2xSSC for 40 seconds, and the slides immersed immediately in cold 70% ethanol for 4 minutes followed by the ethanol series above described. After hybridization for 72 hours and washing, the metaphases were stained with DAPI. Images were captured using the Axiovision 3.0 software with a CCD camera (Axiocam) coupled on a Zeiss-Axiophot 2 microscope or with a software Nis-Elements on a Nikon H550S microscope. For image processing Adobe Photoshop CS4 and GIMP softwares were used.

### Cladistic analysis

Previous results indicated that *Phyllostomus hastatus* karyotype retained most of the segments supposed to be ancestral to family Phyllostomidae [9, 41]. For the sake of convenience, the chromosomal complement of PHA was used as the reference (minimal conserved units) to define all detected segmental associations and/or syntenic disruptions. Structural rearrangements were coded as binary characters and used in a cladistics analysis using maximum parsimony performed with software PAUP4.0b [64]. An exhaustive search with Desmodontinae (DEC, DRO and DYO) [40], as outgroup was performed following the outgroup method choice as suggest by Nixon and Carpenter [65]. All characters had the same weight, based on the premise that chromosome rearrangements have equal chance to occur. A data matrix was established based on the presence or absence of discrete chromosomal homology characters as suggested by Nagamachi et al. [66] and Dobigny et al. [67] (S1 Table). The relative stability of nodes was assessed by bootstrap estimates based on 2,000 iteractions. Each bootstrap replicate involved an exhaustive parsimony search with 10 random taxon additions and tree-bisection-reconnection (TBR) branch swapping. The Bremer support or "decay index" [68, 69] was calculated to verify the inconsistency of the branches in the consensus tree using the software “Tree Analysis Using New Technology” (TNT) version 1.1 (Goloboff et al. [70], freely distributed by the Willi Hennig Society). With the aim to testing the previous phylogenies of these bats using cytogenetics, we compared these results with published molecular topologies on Phyllostominae [5, 15]. We used the chromosome painting to defined the homeologies among the species and then comparing the G bands, being sure that the syntenic blocks are the same [39–41]. Table 1 summarizes the Phyllostomidae species that were compared by chromosome painting.

**Table 1:** Phyllostomidae species compared by chromosome painting in this work.

Subfamily	Species	2n	FN	Cross-species FISH
Micronycterinae	*Micronycteris hirsuta*	25 and 26	32	Ribas et al. 2013
Desmodontinae	*Desmodus rotundus*	28	52	Sotero-Caio et al. 2011
	*Dyameus youngi*	32	60	
	*Dhiphylla eucaudata*	32	60	
Phyllostominae	*Phyllostomus hastatus*	32	58	Pieczarka et al. 2005
	*Phyllostomus discolor*	32	60	This study
	*Lophostoma silvicola*	34	60	This study
	*Tonatia saurophila*	16	20	This study
Carollinae	*Carollia brevicauda*	20/21	36	Pieczarka et al. 2005
Stenodermatinae	*Artibeus obscurus*	30/31	56	Pieczarka et al. 2013
	*Uroderma bilobatum*	42	50	
	*U*. *magnirostrum*	36	62	

2n = diploid number and FN = fundamental number.

## Conclusions

The karyotype of *Tonatia saurophila* is highly derived in relation to that of other phyllostomid bats, differing from the supposed ancestral karyotype of Phyllostomidae by multiple rearrangements. Phylogenies based on chromosomal data are independent evidence for monophyly of tribe Phyllostomini as determined by molecular topologies and provide additional support for the paraphyly of the genus *Tonatia* by the exclusion of the genus *Lophostoma* [5].

## Supporting Information

S1 Table Basic data matrix. Number of characters: 55. Number of informative characters: 17.
(DOCX)
