# Supplementary material for: Phylogenetic Reconstruction by Cross-Species Chromosome Painting and G-Banding in Four Species of Phyllostomini Tribe (Chiroptera, Phyllostomidae) in the Brazilian Amazon: An Independent Evidence for Monophyly
Source: PLoS One. 2015 Mar 25;10(3):e0122845. doi: 10.1371/journal.pone.0122845 (PMC4373847; doi:10.1371/journal.pone.0122845)
Supplement: S1 Table — Number of characters: 55. Number of informative characters: 17. (DOCX) [file pone.0122845.s001.docx]

**Supporting Information**

**Table S1**

**Basic data matrix. Number of characters: 55. Number of informative characters: 17.**

|  | Characters | PHA | LSI | PDI | TSA | DRO | DEC | DYO |
| --- | --- | --- | --- | --- | --- | --- | --- | --- |
| 1 | 1 as in PHA | 1 | 1 | 1 | 0 | 0 | 0 | 0 |
| 2 | Assoc. 1q/3q | 0 | 0 | 0 | 1 | 0 | 0 | 0 |
| 3 | Assoc. 1p inv /12 | 0 | 0 | 0 | 0 | 1 | 1 | 1 |
| 4 | 1p inv free | 0 | 0 | 0 | 0 | 0 | 0 | 1 |
| 5 | Assoc. 1q/10p | 0 | 0 | 0 | 1 | 0 | 0 | 0 |
| 6 | Assoc. 1p/13qd | 0 | 0 | 0 | 1 | 0 | 0 | 0 |
| 7 | Assoc. 1q/5p | 0 | 0 | 0 | 0 | 1 | 1 | 1 |
| 8 | 2 as in PHA | 1 | 1 | 1 | 0 | 0 | 0 | 0 |
| 9 | Assoc. 2p/12p | 0 | 0 | 0 | 1 | 0 | 0 | 0 |
| 10 | Assoc. 2pq prox+5q | 0 | 0 | 0 | 0 | 1 | 1 | 1 |
| 11 | Assoc. 2q+4p | 0 | 0 | 0 | 0 | 1 | 1 | 1 |
| 12 | Assoc. 2q/6p | 0 | 0 | 0 | 1 | 0 | 0 | 0 |
| 13 | 3 as in PHA | 1 | 1 | 1 | 0 | 1 | 1 | 1 |
| 14 | Assoc. 3p/9q | 0 | 0 | 0 | 1 | 0 | 0 | 0 |
| 15 | Assoc. 3q/4pq | 0 | 0 | 0 | 1 | 0 | 0 | 0 |
| 16 | Assoc. 3p/7p | 0 | 0 | 0 | 1 | 0 | 0 | 0 |
| 17 | 4 as in PHA | 1 | 1 | 1 | 0 | 0 | 0 | 0 |
| 18 | 4q inv free | 0 | 0 | 0 | 0 | 1 | 1 | 1 |
| 19 | Assoc. 4p/12p | 0 | 0 | 0 | 1 | 0 | 0 | 0 |
| 20 | Assoc. 4p/13p | 0 | 0 | 0 | 1 | 0 | 0 | 0 |
| 21 | 5 as in PHA | 1 | 1 | 1 | 1 | 0 | 0 | 0 |
| 22 | Assoc. 5/14 | 0 | 0 | 0 | 1 | 0 | 0 | 0 |
| 23 | 6 as in PHA | 1 | 1 | 1 | 0 | 1 | 1 | 1 |
| 24 | Assoc. 6p+7p | 0 | 0 | 0 | 1 | 0 | 0 | 0 |
| 25 | Assoc. 6q/7q | 0 | 0 | 0 | 1 | 0 | 0 | 0 |
| 26 | Assoc. 6p/13p | 0 | 0 | 0 | 1 | 0 | 0 | 0 |
| 27 | Assoc. 6q/11 | 0 | 0 | 0 | 1 | 0 | 0 | 0 |
| 28 | 7 as in PHA | 1 | 1 | 1 | 1 | 0 | 0 | 0 |
| 29 | Assoc. 7q/8p | 0 | 0 | 0 | 1 | 0 | 0 | 0 |
| 30 | 7inv | 0 | 0 | 0 | 0 | 1 | 1 | 1 |
| 31 | 8 as in PHA | 1 | 1 | 1 | 0 | 1 | 1 | 1 |
| 32 | Assoc. 8q/9p | 0 | 0 | 0 | 1 | 0 | 0 | 0 |
| 33 | 9 as in PHA | 1 | 1 | 1 | 0 | 1 | 1 | 1 |
| 34 | Assoc. 9p/13q | 0 | 0 | 0 | 1 | 0 | 0 | 0 |
| 35 | Assoc. 9p/12p/?/10 | 0 | 0 | 0 | 0 | 1 | 0 | 0 |
| 36 | Assoc. 9p/12q/?/15 | 0 | 0 | 0 | 0 | 1 | 0 | 0 |
| 37 | 10 as in PHA | 1 | 1 | 1 | 0 | 1 | 1 | 1 |
| 38 | Assoc. 10p/13qd | 0 | 0 | 0 | 1 | 0 | 0 | 0 |
| 39 | Assoc. 10q/12q | 0 | 0 | 0 | 1 | 0 | 0 | 0 |
| 40 | Assoc. 10q/6p | 0 | 0 | 0 | 1 | 0 | 0 | 0 |
| 41 | 11 as in PHA | 1 | 1 | 1 | 1 | 1 | 1 | 1 |
| 42 | Assoc. 11/15 | 0 | 0 | 0 | 1 | 0 | 0 | 0 |
| 43 | 12 as in PHA | 1 | 1 | 1 | 0 | 0 | 0 | 0 |
| 44 | Assoc. 12p/13inv | 0 | 0 | 0 | 0 | 0 | 0 | 1 |
| 45 | Assoc. 12q/14 | 0 | 0 | 0 | 0 | 0 | 0 | 1 |
| 46 | Assoc. 12/15 | 0 | 0 | 0 | 0 | 0 | 1 | 0 |
| 47 | 13 as in PHA | 1 | 0 | 1 | 0 | 0 | 0 | 0 |
| 48 | PHA-13qdistal free | 0 | 1 | 0 | 0 | ? | ? | ? |
| 49 | 13 p+q prox =(CBR-8) | 0 | 1 | 0 | 0 | 1 | 1 | 1 |
| 50 | Assoc. 13 p+q prox /14 | 0 | 0 | 0 | 0 | 1 | 0 | 0 |
| 51 | Assoc. 13p+q prox/15p | 0 | 0 | 0 | 0 | 0 | 1 | 0 |
| 52 | Assoc. 13q prox/15 | 0 | 0 | 0 | 1 | 0 | 0 | 0 |
| 53 | 14 as in PHA | 1 | 1 | 1 | 1 | 0 | 1 | 0 |
| 54 | 15 as in PHA | 1 | 1 | 1 | 1 | 0 | 0 | 0 |
| 55 | 15inv | 0 | 0 | 1 | 0 | 1 | 1 | 1 |

Note: Assoc=associated; p=short arm; q=long arm; d=distal; prox=proximal; Inv=inversion;

PHA = *Phyllostomus hastatus*; LSI = *Lophostoma silvicola*; TSA = *Tonatia saurophila*; DRO= *Desmodus rotundus*; DEC: *Dhyphylla eucaudata*; DYO = *Diaemus youngi*.
